# Baseline characteristics in stroke patients with atrial fibrillation: clinical trials versus clinical practice

**DOI:** 10.1186/s13104-015-1237-2

**Published:** 2015-06-25

**Authors:** Christian Tanislav, Sonja Milde, Sabine Schwartzkopff, Björn Misselwitz, Nicole Sieweke, Manfred Kaps

**Affiliations:** Department of Neurology, Justus Liebig University, Klinikstrasse 33, 35392 Giessen, Germany; Dresden International University, Dresden, Germany; AOK Division of the Federal State of Hesse, Eschborn, Frankfurt, Germany; Geschäftsstelle Qualitätssicherung Hessen (GQH), Eschborn, Frankfurt, Germany

**Keywords:** Stroke, Oral anticoagulation, Atrial fibrillation, Care delivery, New anticoagulants

## Abstract

**Background:**

When applying information gathered from medical research to the clinical setting, it is imperative that the sample of the investigated patients be representative of the clinical population. Because of this fact, it is necessary to closely examine the sample’s baseline characteristics in clinical trials.

**Methods:**

We analysed baseline data of relevant trials investigating considerable proportions of patients with atrial fibrillation (AF) in the secondary stroke prevention: EAFT, SIFA, Active-W, BAFTA, RE-LY, AVERROES, ARISTOTLE and ROCKET AF. For comparing baseline data stroke patients with AF documented in a statutory stroke registry were considered. In a subgroup of patients (members of a large insurance) data on subsequent prescription for oral anticoagulants (oAK) were available.

**Results:**

In the stroke registry (n = 15,886) the mean age was higher than in the selected clinical trials (mean 77.7 versus 70–72 years). Among insurance members (n = 1,828), those with a prescription for oAK (n = 827) were older than patients recruited in clinical trials (mean 75.1 versus 70–72 years). Results also showed that the male sex was overrepresented in clinical trials (59–63% versus 46%). The distribution of vascular risk factors in recent clinical trials was comparable to proportions in the registry (hypertension: 77–85% versus 80%; diabetes mellitus: 20–26% versus 27%).

**Conclusions:**

The majority of stroke patients with AF in the clinical setting are considerably older than those included in clinical trials. While the distribution of vascular risk factors in clinical trials corresponds to proportions observed in clinical practice, an overrepresentation of the male sex in clinical trials is evident.

## Background

Evidence-based medicine, identified in clinical trials, is crucial to facilitate appropriate decision-making in patient care. However, patients in clinical trials inevitably represent a selected sample of the population. As a result, physicians might challenge the external validity especially in specific subgroups such as elderly patients. Additionally, different sources of bias might distort results. One of the most important and most often discussed confounding variables is selection bias, which occurs when considering patients to participate in a study [1]. Applying information derived from medical research into the clinical setting depends on the generalizability of the investigated patients to the population of interest.

A large number of studies have shown the efficacy of oral anticoagulation (OA) in atrial fibrillation (AF) [2–10]. These studies evaluated primary and secondary stroke prevention with results demonstrating the substantial benefit of OA [2–10]. Therefore, it is crucial to determine how the baseline characteristics of study samples compare to those of patients encountered in clinical practice and nation-wide populations.

In the presented investigation, we aimed to analyse baseline characteristics in relevant clinical trials of atrial fibrillation when compared to stroke victims documented in a larger, more representative stroke registry sample.

## Methods

We evaluated the baseline data of previous clinical trials with substantial proportions of stroke patients with atrial fibrillation, including: EAFT, SIFA, Active-W, BAFTA, RE-LY, AVERROES, ARISTOTLE and ROCKET AF [2–10]. Characteristics such as age, sex, vascular risk factors including hypertension and diabetes mellitus were investigated. For RE-LY, ARISTOTLE and ROCKET AF baseline data and frequencies calculated in the stroke subgroups were collected for analysis.

**Figure 1:**
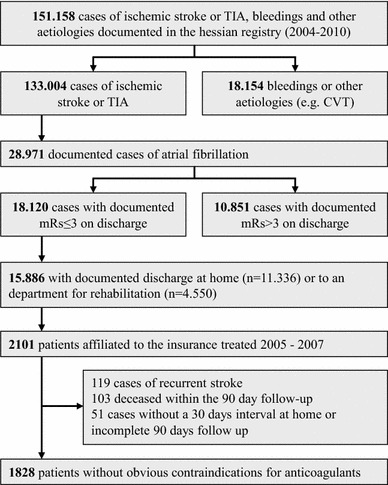
Patient selection within the stroke registry data set. *TIA* refers to transient ischaemic attack, *CVT* refers to cerebral vein thrombosis, *mRs* refers to modified Rankin scale.

For comparison, we gathered baseline data from ischaemic stroke or TIA patients with AF documented (2004–2010) in the stroke registry (n = 15,886) of the Institute of Quality Assurance Hesse (Geschäftsstelle für Qualitätssicherung, GQH) (Figure 1) [11]. Patients with a haemorrhagic stroke or intracranial bleeding were excluded from this study sample. Patients with moderate disability (modified Rankin scale ≤3 as assessed on discharge) were selected because these patients might most meet the criteria for measures in the secondary stroke prevention such as intake of oral anticoagulants. This selection might further provide certain structural equality regarding the disability status when comparing patients in clinical trials versus daily practice.

Among this sample, we selected a subgroup of patients (n = 827) from a large health insurance consortium with evidence of a prescription for oral anticoagulants (oAK). For this subgroup insurance data between 2005 and 2007 was used. In order to identify pertinent members of this insurance, we examined patients matching a set of criteria including date of birth, date of hospital admission and the admitting hospital. This data was linked in a pseudonymous manner. For our analysis, we selected patients without further hospitalisation in a 30-day period within 90 days after discharge (n = 1,828). We defined evidence of a prescription for oral anticoagulants (including phenprocoumaron, warfarin and coumadin) as a marker of anticoagulation. This information was gathered from the insurance claims; 827 patients were identified. For the categorical variables, data was presented in proportions. A Chi squared test was used to compare proportions between the entire sample identified in the Hessian stroke registry, the EAFT and SIFA’s samples, and the subgroups of patients with a previous stroke or TIA in RE-LY, ARISTOTLE and ROCKET AF.

The protocol of the present study was reviewed and approved by the ethical committee of the medical faculty of the Justus Liebig University Giessen.

### GQH

The GQH database is a mandatory nationwide hospital-based registry spanning more than 95% of all ischaemic strokes, transient ischaemic attacks (TIA), and intracerebral haemorrhages in more than 6 million residents of Hesse, Germany. The GQH includes data of acute inpatient treatment, as well as factors proven to be relevant for the course and the prognosis of a stroke. For quality assurance purposes, the acquisition of this data is regulated by law and implemented as a guideline, which is elaborated by the Federal Joint Committee for hospital quality assurance in accordance with Volume V of the Social Insurance Code (§137 SGB V and §135a SGB V). Based on this regulation, the Hesse State Hospital Law contains a provision that allows the GQH to record such data legally. The publication of aggregate quality assurance data has been approved by the Hesse Data Protection Commissioner, so no data protection problem arises here [12–14].

## Results

In the GQH registry sample (n = 15,886) the mean age was 77.7 years. Apart from the BAFTA trial (mean age 81.5 years), the mean age in the selected trials and subgroups of patients with a previous stroke or TIA ranged between 70 and 72 years. In the insurance subgroup the mean age was comparable to the registry sample (77.6 versus 77.7 years) (Table 1). In the insurance subgroup of patients with a prescription for oAK, the mean age was 75.1 years and in those without evidence of a prescription the mean age was 79.8.

**Table 1:** Comparison of baseline characteristics between the registry cohorts versus relevant studies providing evidence on secondary stroke prevention in atrial fibrillation

	Hessian stroke registry (2004–2010) n = 15,886	Insurance (total Cohort) n = 1,828	Insurance (subgroup of patients with prescription for AK) n = 827	EAFT (1993) n = 669	SIFA (1997) n = 916	Active-W (2006) n = 6,706	BAFTA (2007) n = 973	AVERROES (2011) n = 5,599	RE-LY (2010) n = 18,113	ARISTOTLE (2011) n = 18,201	ROCKET AF (2012) n = 14,264
Age (years) mean, ±SD	77.7 (±9)	77.6 (±8)	75.1(± 8)	71 (±7)	72 (± 8)	70.2	81.5 (±8)	70 ± 9	70 (±9)^a^	70.1 (±9.7)^a^	71 (median)^a^ (IQR 64–77)
Sex											
Male	7,312 (46%)	764 (41.8%)	398 (48.1%)	395 (59%)	430 (47%)	4,430 (66%)	531 (55%)	3,277 (59%)	2,279 (63%)^a,†^	2,152 (63%)^a,†^	4,538(61%)^a,†^
Female	8,574 (53%)	1,064 (58%)	429 (51.9%)	274 (41%)	486 (53%)	2,276 (34%)	442 (45%)	2,322 (41%)	1,344 (37%)^a,†^	1,284 (37%)^a,†^	2,930(39%)^a,†^
Risk factors											
Hypertension	12,679 (80%)	1,444 (79%)	660 (79.8%)	294 (44%)	506 (55%)	5,522 (82%)	528 (54%)	4,837 (86%)	2,783 (77%)^a,†^	2,858 (83%)^a,†^	6,343 (85%)^a,†^
Diabetes mellitus	4,371 (27%)	530 (29%)	240 (29%)	87 (13%)	144 (16%)	1,429 (21%)	129 (13%)	1,096 (20%)	816 (22%)^a,†^	902 (26%)^a^	5,695 (24%)^a,†^
Proportion of Strokes or TIAs within the study population	100%	100%	100%	100%	100%	15% (n = 1,006)	13% (n = 124)	14% (n = 764)	20% (n = 3,623)	19% (n = 3,436)	52% (n = 7,468)
Comparison of agents	–	–	–	Warfarin vs. placebo and aspirin vs. placebo	Indobufen^b^ vs. warfarin	Aspirin + clopidogrel vs. warfarin	Warfarin vs. aspirin	Apixaban vs. aspirin	Dabigatran vs. warfarin	Apixaban vs. warfarin	Rivoroxaban vs. warfarin

^a^Data refers to the subgroup of patients with a previous stroke or TIA.

^b^Indobufen is a reversible inhibitor of platelet cyclooxygenase activity.

^†^ *p* value <0.001 calculated with a Chi square test (comparison between the entire cohort determined in the hessian stroke registry (n = 15,886) and the EAFT cohort, the SIFA cohort and the stroke subgroups in RE-LY, ARISTOTLE and ROCKET AF).

The proportion of males in the registry sample was 46%, while in the insurance subgroup only 41% of patients were male. The proportion of males increased in the insurance subgroup of patients with an oAK prescription to 48.1%. Apart from SIFA (proportion of male patients 47%) there was a majority of males reaching 55–66% in the selected clinical trials (Table 1).

The proportion of hypertension and diabetes mellitus in the registry population, in the selected trials, and in the subgroups of patients with a previous stroke or TIA were similar (hypertension 80, 79 and 79.8%); diabetes: 27, 29 and 29%) (Table 1). Earlier investigations (EAFT, SIFA and BAFTA) focused on patients with a moderate vascular burden and had lower proportions of these risk factors (hypertension: 44–55% of the patients; diabetes mellitus: 13–16% of the patients). In recent trials (Active-W, RE-LY, AVERROES, ARISTOTLE and ROCKET AF) these factors are of similar proportions when compared to the registry population (hypertension: 77–86% of the patients; diabetes mellitus: 20–26% of the patients).

Using a Chi squared test, we compared the proportions of these characteristics among the entire study sample, which was composed of the Hessian stroke registry and the EAFT and SIFA populations and the subgroups of patients with stroke or TIA in RE-LY, ARISTORLE and ROCKET AF. Results showed that in the majority of the cases p values were <0.001 (Table 1). However, no relevant differences were identified in the comparison between proportions of patients with diabetes mellitus in the registry study group and the stroke or TIA subgroup in ARISTOTLE (27 versus 26%, *P* = 0.1317) and the gender comparison with SIFA (males: 46 versus 47%, *P* = 0.5889).

## Discussion

In the registry population patients were on average 7 years older than those included in the selected studies [2–10]. Apart from age, the comparison of baseline characteristics revealed an overrepresentation of male individuals in clinical trials. The proportions of vascular risk factors such as hypertension and diabetes mellitus were comparable in recent clinical trials with those seen in clinical practice.

It is also important to note that factors such as age might impact a patient’s inclusion in clinical trials on OA. In these clinical trials, there are strict inclusion criteria that determine whether or not a patient is included. The factor age may bias the inclusion in studies due to the increased risk of bleeding in the elderly and as a result elderly patients may be less selected for inclusion in clinical trials for OA. In our GQH registry sample the mean age within the subgroup of patients with prescriptions for oAK was lower than in the total registry sample (75.1 vs. 77.7 years respectively); however, it still ranged above the mean age observed in clinical trials (75.1 vs. 70–72 years).

The BAFTA trial, which included oAK for individuals with AF over 75 years of age, reported a 2.4% absolute risk reduction per year and a similar risk for major bleeding when compared to aspirin (1.9 versus 2.0%) [8]. While distributions of vascular risk factors in the recent trials are comparable to proportions calculated in the registry population, participants in the BAFTA-trial were less affected by vascular risk factors (hypertension: 80 versus 54%; diabetes mellitus 27 versus 13%) [8]. The burden of leukoaraiosis, microbleeds and silent brain infarctions, which correlates with the presence of vascular risk factors, substantially increases beyond 75 years of age; this results in a higher risk for intracerebral haemorrhage [15–20]. In the BAFTA selection process there was a preference for elderly individuals without considerable vascular burden. Therefore, those patients with supposedly higher risk for bleeding were less preferred for inclusion. Furthermore, regarding secondary stroke prevention, the BAFTA trial is of limited benefit, as only a small proportion of patients who have had a previous stroke were investigated (13%) [8].

ROCKET AF claims to address more elderly patients (median 73 years) [10]. However, in the subgroup of patients with a previous stroke, the median age decreases (median 71 years) corresponding with the subgroups in RE-LY (mean 70.5 years) and ARISTOTLE (mean 70.1 years) [7]. In this case the additional burden of a previous cerebrovascular event a further bias to younger patients.

In many clinical trials males represent the majority of the participants [2–4, 21, 22]. In contrast, in nation-wide data collections, males do not exceed 50% [23–25]. In line with this finding, the proportion of male participants in our nationwide registry was also lower than in clinical trials (46% versus 55–66%). This might also be a result of the increased age of the participants as females suffer from stroke later in life than males [18]. Consistent with this assumption, the slight decrease in age within the insurance subgroups (mean 77.6 versus 75.1 years) corresponded with a higher proportion of male subjects (41.8–48.1%).

Even though EAFT and SIFA included a small number of patients with a relatively moderate burden of vascular risk factors, we considered these two ground-breaking trials, because both specifically addressed stroke patients in secondary prevention [2, 9]. In these trials the significant advantage of oAK over a therapy with platelet inhibitors was demonstrated. In contrast, the vast majority of AF trials provided evidence for the safety and efficacy of OA in the general population of both stroke and non-stroke patients. The percentage of stroke patients ranged from 13 to 25% in these trials. Despite methodological limitations, specifically the small number of patients, these subgroup analyses indicated the efficacy of OA in patients with AF after having a stroke [4, 5, 22]. Indisputably, the participation of stroke patients in AF trials is crucial because they face higher risks of both recurrent stroke and bleeding complications [18]. Because of this higher risk of a worse outcome, it is difficult to extrapolate the benefit of OA in secondary prevention from primary prevention studies. Trials designed to specifically investigate patients within secondary stroke prevention would be ideal.

## Conclusions

Despite clear evidence of the efficacy of OA, there is an age-dependent variable when considering stroke patients for this treatment. There is a considerable difference in age of patient populations in clinical trials when compared with clinical practice. Results have shown that in clinical trials younger patients (70–72 years) are preferable; however, in clinical practice, patients selected for OA are older (75.1 years) than those in trials, but also younger than those who are selected for alternative therapy (79.8 years). With regard to the vascular risk factors of hypertension and diabetes mellitus, we found that the proportions in recent clinical trials are comparable to those in clinical practice. Both patients and clinicians would benefit from further investigations into secondary stroke prevention when selecting patients suitable for OA. Currently evidence is generally collected from studied in the primary stroke prevention with considerably younger patients.

## Abbreviations

OA: Oral anticoagulation
AF: atrial fibrillation
GQH: stroke registry of the Institute of quality assurance Hesse (Geschäftsstelle für Qualitätssicherung, GQH)
TIA: transient ischaemic attacks
oAK: oral anticoagulants
mRS: modified Rankin scale
